# Neuroinflammation and α-Synuclein Dysfunction Potentiate Each Other, Driving Chronic Progression of Neurodegeneration in a Mouse Model of Parkinson’s Disease

**DOI:** 10.1289/ehp.1003013

**Published:** 2011-01-18

**Authors:** Hui-Ming Gao, Feng Zhang, Hui Zhou, Wayneho Kam, Belinda Wilson, Jau-Shyong Hong

**Affiliations:** Laboratory of Toxicology and Pharmacology, National Institute of Environmental Health Sciences, National Institutes of Health, Department of Health and Human Services, Research Triangle Park, North Carolina, USA

**Keywords:** α-synuclein, gene-environment interaction, inflammation, iNOS, microglia, NADPH oxidase, neurodegeneration, oxidative stress, Parkinson’s disease

## Abstract

**Background:**

Mechanisms whereby gene–environment interactions mediate chronic, progressive neurodegenerative processes in Parkinson’s disease (PD)—the second most common neurodegenerative disease—remain elusive.

**Objective:**

We created a two-hit [neuroinflammation and mutant α-synuclein (α-syn) overexpression] animal model to investigate mechanisms through which mutant α-syn and inflammation work in concert to mediate chronic PD neurodegeneration.

**Methods:**

We used an intraperitoneal injection of the inflammogen lipopolysaccharide (LPS; 3 × 10^6^ EU/kg) to initiate systemic and brain inflammation in wild-type (WT) mice and transgenic (Tg) mice overexpressing human A53T mutant α-syn. We then evaluated nigral dopaminergic neurodegeneration, α-syn pathology, and neuroinflammation.

**Results:**

After LPS injection, both WT and Tg mice initially displayed indistinguishable acute neuroinflammation; however, only Tg mice developed persistent neuroinflammation, chronic progressive degeneration of the nigrostriatal dopamine pathway, accumulation of aggregated, nitrated α-syn, and formation of Lewy body-like inclusions in nigral neurons. Further mechanistic studies indicated that 4-week infusion of two inhibitors of inducible nitric oxide synthase and NADPH oxidase, major free radical–generating enzymes in activated microglia, blocked nigral α-syn pathology and neurodegeneration in LPS-injected Tg mice.

**Conclusions:**

Microglia-derived oxidative stress bridged neuroinflammation and α-syn pathogenic alteration in mediating chronic PD progression. Our two-hit animal model involving both a genetic lesion and an environmental trigger reproduced key features of PD and demonstrated synergistic effects of genetic predisposition and environmental exposures in the development of PD. The chronic progressive nature of dopaminergic neurodegeneration, which is absent in most existing PD models, makes this new model invaluable for the study of mechanisms of PD progression.

Parkinson’s disease (PD), an age-related neurodegenerative disorder, progresses over decades. The mechanism for the gradual loss of nigral dopamine (DA) neurons and the chronic disease progression in PD is largely unknown. Emerging evidence has indicated that neuroinflammation contributes to PD (Block et al. 2007; Gao and Hong 2008; Hirsch and Hunot 2009). Morphologic changes of microglia (the resident brain immune cells) and accumulation of proinflammatory factors are associated with degenerating DA neurons in the substantia nigra (SN) of PD patients and various PD animal models (Liberatore et al. 1999; McGeer et al. 1988; Sherer et al. 2003). DNA polymorphisms of several inflammatory cytokines and genetic variation in the HLA (human leukocyte antigen) region that contains numerous immune-related genes might increase the risk of PD (Hamza et al. 2010; Wahner et al. 2007). Moreover, inhibition of inflammation is associated with reduced neuronal impairment in various PD models (Gao et al. 2003; Wu et al. 2003). We and others previously reported that an intraperitoneal injection or chronic nigral infusion of relatively high doses of lipopolysaccharide (LPS; a common inflammogen) led to time-dependent degeneration of nigral DA neurons in rodents (Gao et al. 2002; Ling et al. 2006; Qin et al. 2007). However, mechanisms through which an acute neuroinflammatory response is transformed into a chronic inflammatory process, and chronic neuroinflammation influences progressive neurodegeneration, require further investigation.

Numerous mutations in multiple genes have been identified in familial PD. α-Synuclein (α-syn) is closely associated with both familial and sporadic PD. Missense mutations (A53T, A30P, and E46K) and multiplications of the α-syn gene (*SNCA*) cause autosomal-dominant PD (Farrer 2006; Krüger et al. 1998; Polymeropoulos et al. 1997). Disease severity in PD patients with *SNCA* multiplications positively correlates with gene copy number (Chartier-Harlin et al. 2004; Ibanez et al. 2004; Singleton et al. 2003). Common variability in *SNCA* has been associated with a greater risk of sporadic PD (Pals et al. 2004). Additionally, age-related increases of α-syn in monkeys and humans are linked to nigrostriatal DA depletion (Chu and Kordower 2007). Furthermore, α-syn aggregates are the primary component of Lewy bodies in both familial and sporadic PD (Spillantini et al. 1997). The occurrence of α-syn–positive inclusions in neurons grafted into the brains of PD patients and a transgenic (Tg) PD model (Desplats et al. 2009; Kordower et al. 2008; Li et al. 2008; Mendez et al. 2008) further emphasizes a pivotal role of α-syn in ongoing disease progression and host-to-graft disease propagation.

The current consensus suggests that PD develops from complex gene–environment interactions. For the present study, we created a two-hit, chronic, progressive model that we used to investigate mechanisms of PD progression. Specifically, we examined how mutant human α-syn and low-grade neuroinflammation interacted and influenced PD neurodegeneration and disease progression in Tg mice overexpressing A53T mutant human α-syn. We intraperitoneally injected a relatively low dose of LPS (3 × 10^6^ EU/kg) to induce systemic inflammation that transferred into the brain to form a comparatively mild neuroinflammatory response.

## Materials and Methods

### Animals and the LPS injection

Male homozygous α-syn Tg mice and B6C3F1/J mice [wild-type (WT) controls] were obtained from the Jackson Laboratory (Bar Harbor, ME) and treated humanely and with regard for alleviation of suffering following the *NIH Guide for Care and Use of Laboratory Animals* (Institute of Laboratory Animal Resources 1996). The Tg mice were generated on a C57BL/C3H background and expressed endogenous α-syn and human A53T mutant α-syn driven by a mouse prion protein promoter (Giasson et al. 2002). Seven-month-old WT and Tg mice received an intraperitoneal injection of normal saline (NS) or LPS (*Escherichia coli* 0111:B4; 3 × 10^6^ EU/kg). Four-week infusion of 1400W [an inhibitor of inducible nitric oxide synthase (iNOS); 50 μg/kg/hr] and diphenyleneiodonium (DPI; a NADPH oxidase inhibitor; 5 μg/kg/hr) or vehicle (phosphate-buffered saline containing 6% DMSO) via osmotic minipumps (0.25 μL/hr; 90 μL total capacity; replaced once) was started 30 min before the intraperitoneal injection of NS/LPS. None of the Tg mice presented overt neurologic impairment at the time of LPS/NS injection. Mice were euthanized at desired time points in an age-matched fashion.

### Immmunohistochemistry, confocal double-label immunofluorescence, and cell counting

We used the following primary antibodies for immunostaining, confocal double-label immunofluorescence, and cell counts: SYN211 (1:500; Millipore, Billerica, MA), nSYN514 (1:150; Santa Cruz Biotechnology, Santa Cruz, CA), and antibodies against a neuron-specific nuclear protein (Neu-N; 1:2,000; Chemicon, Billerica, MA), tyrosine hydroxylase (TH; 1:1,000; Sigma Chemical Company, St. Louis, MO), or ionized calcium binding adaptor molecule-1 (Iba-1; 1:500; Wako Chemicals, Richmond, VA). For details of immunostaining assays and counting of immunostained cells, see Supplemental Material (doi:10.1289/ehp.1003013). Briefly, we incubated 30-μm brain sections with 99% formic acid for 5 min for antigen retrieval before performing double-label immmunohistochemistry using nSYN514 and Neu-N antibodies. Sections were then incubated with Alexa-488 (green) and Alexa-594 (red) conjugated secondary antibodies (1:1,000), both from Invitrogen (Carlsbad, CA). We counterstained the nuclei with DAPI (4′,6-diamidino-2-phenylindole). To monitor DA neurodegeneration, two individuals blind to the treatment counted the number of TH-immunoreactive (TH-IR) neurons in the SN pars compacta (SNpc) of six evenly spaced brain sections from a series of 24 sections that covered the entire SN (Zhang et al. 2004).

### Sequential biochemical fractionation and Western blotting

Sequential protein extraction initially removed highly soluble proteins using high-salt (HS) buffer. Proteins from insoluble pellets were then extracted using buffers with increasing solubilization strength: HST buffer (HS buffer containing 1% Triton X-100), RIPA (radioimmunoprecipitation assay) buffer, and 2% sodium dodecyl sulfate (SDS). For all other biochemical studies, we used the 2% SDS buffer to extract both soluble and aggregated proteins from stratum, SN, or whole brains (Gao et al. 2008; Giasson et al. 2002).

For Western blotting, we used the following primary antibodies: SYN211 (1:1,000), nSYN514 (1:250), α-synuclein (C-20)-R (reactive to mouse, rat, and human α-syn; Santa Cruz Biotechnology), or antibodies specific for iNOS (1:500), macrophage antigen complex 1 (Mac1; 1:1,000), TH (1:5,000), Neu-N (1:500), cyclooxygenase-2 (COX-2; 1:1,000), or gp91^phox^ (1:2,000). We included monoclonal anti-β-actin antibody (1:5,000) as an internal standard to monitor loading errors (Gao et al. 2008; Giasson et al. 2002). See Supplemental Material (doi:10.1289/ehp.1003013) for details.

### Statistical analysis

All values are expressed as mean ± SE. Differences among means were analyzed using one- or two-way analysis of variance (ANOVA) with treatment or genotype as independent factors. When ANOVA showed significant differences, pairwise comparisons between means were tested by Newman-Keuls post hoc testing. In all analyses, the null hypothesis was rejected at the 0.05 level.

## Results

### Delayed progressive nigral neurodegeneration in α-Syn Tg mice, but not WT mice, after LPS challenge

**Figure 1:**
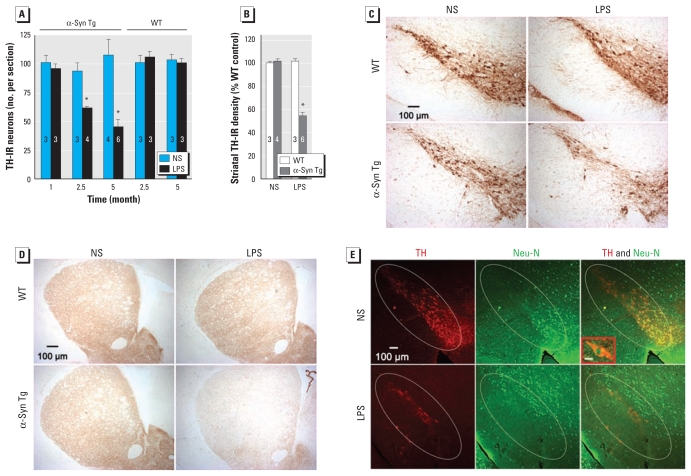
α-Syn Tg mice were more sensitive to inflammation-mediated neurotoxicity. (*A*) Time-dependent loss of nigral DA neurons in α-syn Tg mice, but not WT mice, after LPS injection. (*B*) Densitometry analysis revealed a 46% decrease in striatal TH immunostaining in Tg mice 5 months after LPS injection. In *A* and *B,* numbers within the bars indicate the number of mice in each group at each time point. (*C* and *D*) Representative brain images with TH immunostaining 5 months after LPS injection indicate prominent dopaminergic lesions of SN (*C*) and striatum (*D*) in Tg mice but not in WT mice. (*E*) In the SN of LPS-injected Tg mice, the loss of DA neurons (double-labeled with anti-TH antibody in cytoplasm and anti–Neu-N antibody in nuclei) 5 months after LPS was greater than that of non-DA neurons (green nuclear Neu-N staining only). The inset shows a double-labeled DA neuron. In inset, bar = 10 μm. *p < 0.05 compared with the corresponding NS-injected control.

Both α-syn and neuroinflammation are implicated in PD, but it is unclear how their interaction affects chronic progression of PD. Additionally, for unknown reasons, most available α-syn Tg mice fail to develop overt nigral DA neurodegeneration while exhibiting widespread α-syn pathology in the central nervous system (Giasson et al. 2002). We therefore created a chronic, two-hit mouse model to investigate whether inflammation enhances α-syn pathogenic alteration and accelerates neurodegeneration and disease progression. After LPS injection, Tg mice, but not WT mice, developed delayed, chronic and progressive degeneration of nigral DA neurons, as shown by time-dependent decreases in the number of TH-IR neurons (Figure 1A–D). In particular, 5 months after LPS injection, Tg mice lost 58% of nigral DA neurons (Figure 1A), whereas their striatal TH levels were reduced by 46% (Figure 1B). In contrast, neither nigral DA neurons nor striatal TH levels were altered significantly in LPS-injected WT mice compared with NS-injected WT mice. We identified synergistic neurotoxicity of LPS treatment and α-syn overexpression when the difference in cell loss between NS-injected WT mice and LPS-injected Tg mice was significantly greater than the sum of cell loss observed in NS-injected Tg mice (i.e., mice with the genotype but not the environmental exposure) and LPS-injected WT mice (i.e., mice with the environmental exposure only). TH immunostaining demonstrated the destruction of nigral DA neurons and striatal DA fibers in LPS-injected Tg mice, whereas the nigrostriatal pathway in NS-injected Tg mice and all WT mice remained intact (Figure 1C,D). The relative selectivity of nigral neurodegeneration was characterized by double-label immunofluorescence (Figure 1E). In the SNpc of LPS-injected Tg mice, the number of DA neurons (TH^+^/Neu-N^+^) was decreased by 52 ± 5.6%, whereas non-DA neurons (TH^−^/Neu-N^+^) were relatively spared, as indicated by a 9.2 ± 4.9% reduction. Here, the average number of DA neurons (TH^+^/Neu-N^+^) and non-DA neurons (TH^−^/Neu-N^+^) per SNpc region in the NS-injected Tg was 96 ± 7.4 and 51 ± 9.1, respectively. Moreover, Western blotting (Figure 5) showed that in LPS- versus NS-injected Tg mice, there was a 53% reduction in nigral TH (indicating loss of DA neurons) and a 20% reduction in nigral Neu-N (indicating loss of DA neurons and probably some non-DA neurons in the SN) 5 months after LPS administration. Consistent with the pattern of neuronal loss observed in PD patients, the destruction of nigral DA neurons was not accompanied by significant damage to DA neurons in the adjacent ventral tegmental area. The numbers of TH-IR neurons in the ventral tegmental area of Tg mice 5 months after the injection were 159 ± 8.19 (NS; *n* = 4) and 154 ± 5.18 (LPS; *n* = 4). Collectively, this two-hit PD model reproduced the signature lesion of PD, the relatively selective degeneration of DA neurons in the SN.

### Accumulation and pathologic modification of insoluble, aggregated α-syn in LPS-injected Tg mice

**Figure 2:**
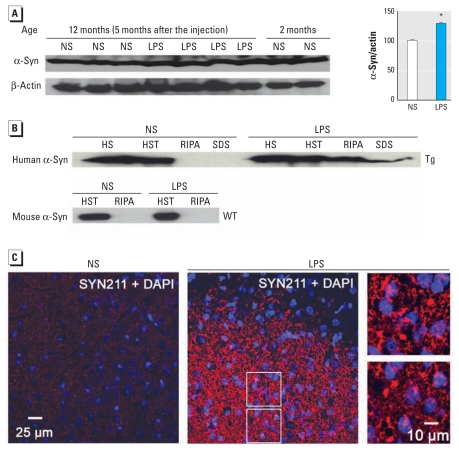
Accumulation of insoluble and aggregated α-syn after LPS injection. (*A*) Five months after treatment, LPS-injected Tg mice accumulated more α-syn in the whole brain than did NS-injected Tg mice, as shown by immunoblotting using SYN211 antibody (specific for human α-syn) and summarized in the graph. (*B*) Sequential extraction detected RIPA-insoluble α-syn in midbrains of LPS-injected Tg mice; α-syn of WT mice was recovered only in HS- and HST-soluble fractions. In *A* and *B*, each lane is the most representative sample from among at least three different animals. (*C*). In contrast with diffuse and faint α-syn staining observed in the SN of NS-injected Tg mice 5 months after the injection (left), α-syn (red) in LPS-injected Tg mice (center) appeared mainly in perinuclear locations and formed aggregates; magnifications (right) are from areas indicated in the LPS section (center). *p < 0.05 compared with the NS-injected control.

In addition to developing progressive neurodegeneration, LPS-injected Tg mice accumulated more α-syn in the whole brain 5 months after injection than did NS-injected Tg mice (Figure 2A). Next, sequential extraction using buffers with increasing solubilization strength detected highly insoluble α-syn in midbrain SDS fractions of Tg mice 5 months after LPS injection. By contrast, midbrain α-syn of NS-injected Tg mice and age-matched WT mice injected with NS/LPS was soluble in HS and HST buffers (Figure 2B). Unlike midbrains, the cortex and spinal cord of 12-month-old Tg mice displayed age-related accumulation of insoluble α-syn in the absence of any treatment (data not shown), which was in accordance with earlier observations (Giasson et al. 2002).

**Figure 3:**
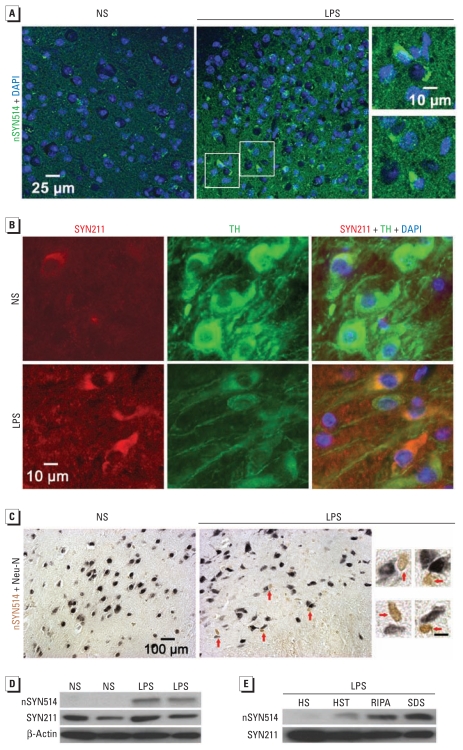
Nitration of aggregated α-syn and formation of cytoplasmic inclusions in Tg mice 5 months after LPS injection. (*A*) Nitrated human α-syn (labeled with nSYN514 antibody) formed aggregates and was mainly perinuclear in the SN of LPS-injected Tg mice (center), whereas nSYN514 staining in NS-injected Tg mice (left) was scant. Magnifications (right) are from areas shown in the LPS section. (*B*) The SN of LPS-injected Tg mice contained human α-syn aggregates (red) in TH-IR neurons (green). (*C*) Cytoplasmic inclusions containing nitrated human α-syn (brown) in nigral neurons (with nuclei stained in dark blue) indicated by red arrows in LPS-injected Tg mice (center) and magnified photomicrographs (right) from the LPS section. The SN of NS-injected Tg mice (left) did not exhibit obvious staining for nitrated α-syn. (*D*) Western blotting shows nitrated human α-syn in midbrain extracts from LPS-injected Tg mice but not NS-injected Tg mice. (*E*) The accumulation of nitrated human α-syn was observed primarily in RIPA and SDS fractions from midbrains of Tg mice. At least four mice in each group were used for immunostaining and immunoblotting. For magnifications in *C*, bars = 10 μm.

Immunofluorescence analysis revealed accumulation of α-syn–positive aggregates in perinuclear locations in the SN of LPS-injected Tg mice (Figure 2C). nSYN514 antibody (specific for nitrated human α-syn) positively stained these aggregates (Figure 3A). Double-label immunofluorescence demonstrated α-syn aggregates in TH-IR neurons in the SN of LPS-injected Tg mice (Figure 3B). Moreover, nitrated α-syn accumulated in neurons to form cytoplasmic inclusions (Figure 3C). In contrast, nigral α-syn immunoreactivity in NS-injected Tg mice was diffuse and barely visible (Figures 2C and 3A–C). In midbrains of LPS-injected but not NS-injected Tg mice, immunoblotting also detected nitrated human α-syn, which appeared predominantly in HS- and HST-insoluble fractions (Figure 3D,E). Thus, in this two-hit model, LPS-induced inflammation accelerated α-syn accumulation, aggregation, and nitration.

### Similar acute systemic and brain inflammatory reactions in WT and Tg mice after LPS injection

To determine the basis for differential nigral α-syn pathology and neurodegeneration in Tg and WT mice after LPS exposure, we first examined acute inflammation in these mice. Tg and WT mice reacted to LPS in an indistinguishable way. Serum tumor necrosis factor-α (TNF-α) and interleukin-1β (IL-1β) were greatly increased to similar levels after LPS injection in both genotypes, implying that genotype did not alter acute systemic inflammation in response to LPS [see Supplemental Material, Figure 1A (doi:10.1289/ehp.1003013)]. We chose TNF-α and IL-1β as representative indicators of systemic inflammation because both cytokines are crucial for systemic effects of inflammation, including fever, increased heart rate, and sepsis, and because TNF-α was one of the major proinflammatory cytokines responsible for transferring peripheral inflammation to the brain when LPS was intraperitoneally injected into mice (Qin et al. 2007).

The absence of detectable differences between WT and Tg mice in the expression of Mac1 and Iba-1 in the brain 1 day after LPS injection suggests similar acute neuroinflammatory responses in both genotypes. Microglia in the SN displayed similar acute morphologic transformation from resting to activated states in both types of mice [see Supplemental Material, Figure 1B–D (doi:10.1289/ehp.1003013)]. Thus, the acute systemic and brain inflammatory reactions to LPS were not distinguishably different in Tg and WT mice.

### Association of α-Syn pathology and progressive nigral neurodegeneration with prolonged neuroinflammation

**Figure 4:**
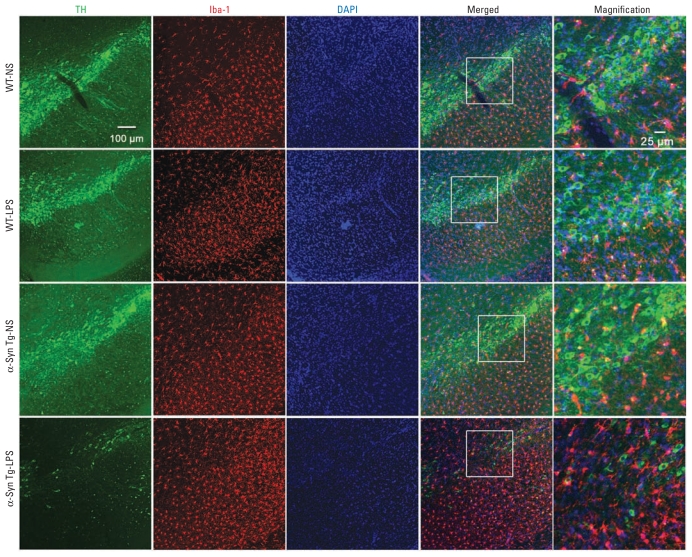
Association between progressive nigral neurodegeneration and prolonged neuroinflammation. Double-label immunofluorescence shows loss of TH-positive DA neurons and degeneration of DA fibers in the SN of α-syn Tg mice 5 months after LPS injection. Only in LPS-injected Tg mice did microglia show active morphology: elevated expression of Iba-1 (red), larger size, irregular shape, and increased numbers in the SN. Microglia of WT mice exhibit resting ramified morphology. Magnifications (right) are from areas indicated in the merged column. All images are representative samples from among at least three different animals in each group.

**Figure 5:**
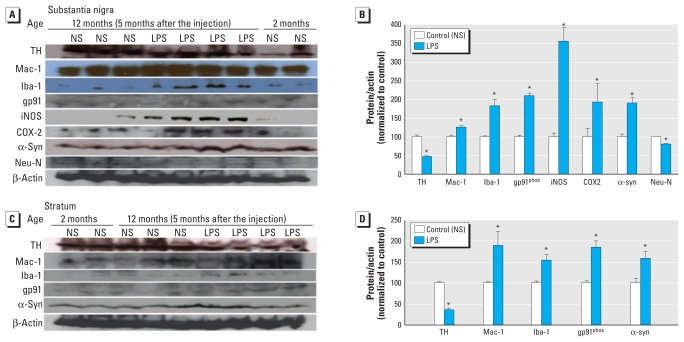
Association of neurodegeneration with chronic α-syn accumulation and prolonged neuroinflammation. Five months after LPS injection, Tg mice displayed significant decreases in TH levels, marked up-regulation of multiple inflammatory markers (Mac1, Iba-1, gp91, iNOS, and COX-2), and accumulation of α-syn in the SN (*A* and *B*) and striatum (*C* and *D*). NS-injected 12-month-old Tg mice did not show overt changes in levels of TH, α-syn, or inflammatory markers, in contrast with 2-month-old Tg mice. Graphs (*B* and *D*) show the ratio of densitometry values (mean ± SE) of each examined protein, and β-actin was normalized to each respective NS-injected control. All immunoblot lanes are representative samples from among at least three different animals in each group. *p < 0.05 compared with corresponding NS-injected control mice.

We next detected chronic nigral neuroinflammation in Tg mice, but not WT mice (Figure 4). Five months after LPS injection, nigral microglia of Tg mice were activated, exhibiting enlarged cell bodies, elevated expression of Iba-1, and increased density in the SN. Microglia of LPS-injected WT mice revealed ramified resting morphology; levels of Iba-1 expression in microglia were similar in LPS-injected and NS-injected WT mice (Figure 4). Five months after LPS exposure, we observed marked up-regulation of multiple inflammatory markers, including Iba-1, Mac1, iNOS, COX-2, and gp91^phox^, in both the SN and striatum of Tg mice, indicating prolonged neuroinflammation in these regions (Figure 5).

By repeatedly probing, stripping, and reprobing the same immunoblotting membranes, we observed that the persistent neuroinflammation was negatively correlated with TH levels and positively correlated with α-syn levels in both the SN and striatum of LPS-injected Tg mice (Figure 5). In concurrence with elevated inflammatory mediators, α-syn levels in the SN and striatum showed 80% and 60% increases, respectively, and TH levels were decreased by approximately 50% (Figure 5). The more pronounced increase in α-syn expression in the SN and striatum (60–80%) than in the whole brain (28%; Figure 2A) of LPS-challenged Tg mice may be attributable, at least partially, to the high density of microglia (Kim et al. 2000) and more marked inflammatory responses in these brain regions.

Unlike LPS-injected Tg mice, NS-injected Tg mice and LPS-injected WT mice failed to develop chronic neuroinflammation (Figure 4), α-syn pathology (Figures 2 and 3), or detectable neuronal loss (Figures 1, 4, and 5) in the nigrostriatal pathway. These data together demonstrate that chronic progression of PD was driven by synergistic effects of low-grade neuroinflammation and α-syn dysfunction. In contrast, neither factor appeared to be sufficient to cause neuronal death in isolation.

### Continuous inhibition of iNOS and NADPH oxidase blocked α-syn pathology and nigral neurodegeneration

**Figure 6:**
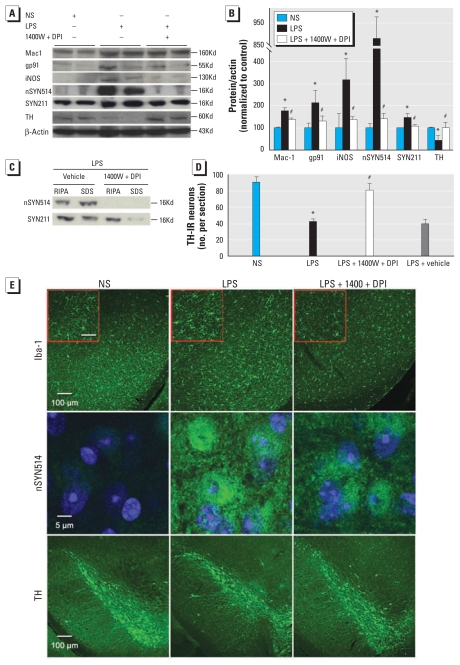
Inhibition of iNOS and NADPH oxidase prevent chronic dopaminergic neurodegeneration and α-syn pathology in LPS-injected Tg mice. (*A*) Immunoblotting analysis of midbrain proteins extracted by 2% SDS buffer indicates that a 4-week infusion of 1400W and DPI blunted up-regulation of inflammatory markers (Mac1, iNOS, and gp91^phox^), attenuated loss of TH, and reduced accumulation of nitrated α-syn in Tg mice 3 months after LPS injection. (*B*) Densitometry analysis of proteins and β-actin examined in *A*; Results are mean ± SE normalized to the corresponding NS-injected control. (*C*) Accumulation of nitrated human α-syn with low solubility in RIPA and SDS fractions from midbrains of Tg mice 3 months after LPS injection, which was blocked by 1400W and DPI. (*D*) Blockage of chronic degeneration of nigral DA neurons in α-syn Tg mice 3 months after LPS injection by 1400W and DPI. (*E*) Representative brain images of α-syn Tg mice 3 months after LPS indicate that a 4-week infusion of 1400W and DPI dampened chronic microglial activation, attenuated accumulation of α-syn, and reduced nigral DA lesions. Insets are magnified photomicrographs of corresponding images. In insets, bar = 25 μm. For immunoblotting and immunostaining, *n* = 4 mice per group. *p < 0.05 compared with corresponding NS-injected control mice; ^#^*p* < 0.05 compared with corresponding LPS-injected mice.

NADPH oxidase and iNOS are major free radical–generating enzymes in activated microglia. The marked up-regulation of iNOS and gp91^phox^ (the catalytic subunit of NADPH oxidase) in the nigrostriatal pathway of LPS-injected Tg mice (Figure 5A,B) prompted us to hypothesize that microglia-derived free radicals (e.g., superoxide, nitric oxide, and their reaction product peroxynitrite) caused the nitration of α-syn, which in turn promoted formation of α-syn aggregates and sensitized DA neurons to diverse insults. Inhibition of NADPH oxidase and iNOS by 4-week infusion of DPI and 1400W reduced chronic neuroinflammation (Figure 6A,B,E), blocked nigral α-syn pathology (Figure 6A–C,E), and prevented dopaminergic neurodegeneration (Figure 5A,B,D,E) in Tg mice 3 months after LPS exposure. Within the brain, both iNOS and NADPH oxidase are primarily located in microglia. Therefore, the attenuation of Mac1, gp91^phox^, and iNOS up-regulation by 1400W and DPI implies that microglia-derived free radicals played a critical role in maintaining chronic neuroinflammation (Figure 6A,B). The correlation of dampened neuroinflammation with abolished nitration of α-syn, reduced accumulation of insoluble α-syn, and attenuated neuronal impairment is consistent with a causative relationship between inflammation-mediated α-syn pathologic alterations and chronic dopaminergic neurodegeneration.

## Discussion

The present study demonstrates that persistent neuroinflammation bridges α-syn pathologic alterations and progressive neurodegeneration in mediating chronic PD progression. The two-hit (neuroinflammation and mutant α-syn overexpression) progressive animal model created for this study mimics PD multifactorial etiology and reproduces the following key features of PD: *a*) chronic, progressive, and relatively selective degeneration of dopaminergic neurons and fibers in the nigrostriatal pathway; *b*) Lewy body-like neuronal inclusions containing aggregated α-syn; *c*) persistent neuroinflammation, a common feature shared by all neurodegenerative diseases including PD; and *d*) a chronic progressive disease course that is absent in most available PD models. These key features of PD were replicated in LPS-injected α-syn Tg mice, but not in NS-injected Tg mice or in LPS-injected WT mice, indicating synergistic effects of environmental exposures and genetic predisposition in the pathogenesis of PD.

α-Syn plays a prominent role in PD onset, ongoing disease progression, and host-to-graft disease propagation. In general, Tg overexpression of human α-syn results in widespread α-syn pathologies and variable neurodegeneration in different animal species (Farrer 2006; Lee et al. 2002). Among different α-syn Tg mouse models, only a few exhibit overt degeneration of nigrostriatal DA fibers and even fewer show loss of nigral DA neurons (Dawson et al. 2010; Lee et al. 2002; Masliah et al. 2000). The lack of DA neuron loss in most gene-based PD animal models and the dearth of α-syn pathology in most toxin-based PD animal models underscore a crucial role of gene–environment interactions in PD. The low rate of concordance for PD in monozygotic and dizygotic twins (Marttila et al. 1988; Tanner 2003) strongly suggests the involvement of environmental factors in PD. Epidemiologic and case–control studies have implicated rural living, well water consumption, and pesticide exposure as potential environmental risk factors for PD (Elbaz et al. 2009). Interestingly, it has been reported that overexpression of mutant α-syn in mice protects against (Manning-Bog et al. 2003) or fails to affect paraquat neurotoxicity (Norris et al. 2007), augments neurodegeneration elicited by 1-methyl-4-phenyl-1,2,3,6-tetrahydropyridine and LPS (Gao et al. 2008; Nieto et al. 2005), or does not enhance rotenone-induced neurotoxicity (Nieto et al. 2005). These findings indicate that interactions between α-syn and environmental toxins on PD neurodegeneration require further investigation. Another newly developed two-hit mouse model combining a genetic lesion (Parkin^−/−^) and neuroinflammation has provided additional experimental evidence supporting a crucial role of gene–environment interactions in PD (Frank-Cannon et al. 2008).

Compared with a previous mouse model, generated by an intraperitoneal injection of a higher dose of LPS (5 mg/kg, equal to ~ 15 × 10^6^ EU/kg) into WT mice (Qin et al. 2007), our two-hit model created by a moderately toxic dose of LPS (3 × 10^6^ EU/kg) revealed accelerated DA neurodegeneration and provided additional insight into α-syn pathologic alterations under inflammatory states. Specifically, significant loss of DA neurons started to manifest at 7 months after LPS injection in the prior model (Qin et al. 2007) and at 2.5 months in our two-hit model; at these two time points, a 23% and 37% loss of DA neurons was observed, respectively. A single nigral injection of LPS resulted in acute, dramatic nigral neuroinflammation and DA neuron loss in mutant α-syn Tg mice (Gao et al. 2008), but that acute two-hit model did not replicate the chronic, progressive course of PD, and acute, robust, and localized nigral inflammation may not be highly relevant to potential etiologies or pathogenesis of PD. The present study therefore advances research to elucidate a mechanistic link among persistent neuroinflammation, chronic α-syn pathology, and progressive neurodegeneration in chronic PD progression.

Persistent activation of iNOS and NADPH oxidase in LPS-treated Tg mice (Figure 5) and blockage of nitrated α-syn formation by continuous inhibition of these enzymes (Figure 6) indicated that increased nitric oxide, superoxide, and peroxynitrite may nitrosylate and/or oxidize α-syn and lower its solubility to form aggregates (Figures 2 and 3). Aggregated α-syn may overwhelm the ubiquitin–proteasome system and accumulate over time, leading to endoplasmic reticulum stress and mitochondrial impairment (Cooper et al. 2006). Loss of physiologic function of normal α-syn and oxidative insults from microglia-derived free radicals further exacerbate neuronal damage, eventually causing neuronal death. DA neurons in the SN are known to be uniquely vulnerable to oxidative insults (Jenner and Olanow 1998), and they encounter an excessively high level of oxidative stress during brain inflammation because of the high density of microglia in this region (Kim et al. 2000). The combination of these factors may be partially responsible for the relatively selective degeneration of DA neurons in the SN in PD.

We have proposed that dying/dead neurons further activate microglia by releasing noxious compounds (e.g., α-syn) into the extracellular milieu (Gao and Hong 2008). Indeed, monomeric and aggregated α-syn can be released from cultured cells and is recovered from the cerebrospinal fluid of patients with PD (Liu et al. 2009). Exogenous application of aggregated α-syn activates cultured microglia (Zhang et al. 2005). Therefore, ongoing neurodegeneration and chronic neuroinflammation cause a vicious self-propelling cycle that makes it possible for neuroinflammation to persist long after peripheral inflammation has abated, and for neurodegeneration to become a chronic progressive process. Without positive feedback from damaged neurons, the low-grade acute neuroinflammation induced by LPS was not sustained in WT mice, and consequent effects on α-syn pathology and neurodegeneration were negligible.

## Conclusions

Our two-hit progressive model underscores a central role of gene–environment interactions in the development of PD. The attenuation of nigral α-syn pathology and dopaminergic neurodegeneration by the inhibition of inflammatory enzymes—NADPH oxidase and iNOS—strongly supports a causative relationship between inflammation-mediated α-syn pathologic alterations and chronic dopaminergic neurodegeneration. Microglia-derived free radicals were major factors that bridged neuroinflammation and α-syn dysfunction in mediating chronic PD neurodegeneration.
